# Knowledge Development Trajectories of Intelligent Video Surveillance Domain: An Academic Study Based on Citation and Main Path Analysis

**DOI:** 10.3390/s24072240

**Published:** 2024-03-31

**Authors:** Fei-Lung Huang, Kai-Ying Chen, Wei-Hao Su

**Affiliations:** 1 Department of Industrial Engineering & Management, National Taipei University of Technology, Taipei 10608, Taiwan; mikehuang168@gmail.com (F.-L.H.); kychen@mail.ntut.edu.tw (K.-Y.C.); 2 Department of Transportation Science, National Taiwan Ocean University, No. 2, Beining Rd., Zhongzheng Dist., Keelung City 202301, Taiwan

**Keywords:** intelligent video surveillance, security, video analytics, privacy preservation, Internet of things, main path analysis

## Abstract

Smart city is an area where the Internet of things is used effectively with sensors. The data used by smart city can be collected through the cameras, sensors etc. Intelligent video surveillance (IVS) systems integrate multiple networked cameras for automatic surveillance purposes. Such systems can analyze and monitor video data and perform automatic functions required by users. This study performed main path analysis (MPA) to explore the development trends of IVS research. First, relevant articles were retrieved from the Web of Science database. Next, MPA was performed to analyze development trends in relevant research, and g-index and h-index values were analyzed to identify influential journals. Cluster analysis was then performed to group similar articles, and Wordle was used to display the key words of each group in word clouds. These key words served as the basis for naming their corresponding groups. Data mining and statistical analysis yielded six major IVS research topics, namely video cameras, background modeling, closed-circuit television, multiple cameras, person reidentification, and privacy, security, and protection. These topics can boost the future innovation and development of IVS technology and contribute to smart transportation, smart city, and other applications. According to the study results, predictions were made regarding developments in IVS research to provide recommendations for future research.

## 1. Introduction

The COVID-19 pandemic accelerated the advancement of intelligent video surveillance (IVS) technologies with capabilities in mask detection, temperature measurement, and crowd analysis. These advancements align with the global trend toward the establishment of smart cities, in which video data plays a crucial role in enhancing urban governance efficiency. Additionally, growing concerns over safety and security have led to an increased demand for IVS technologies due to increasing crime rates. Accordingly, the global video surveillance market is expected to reach US$53.7 billion by 2023 and US$83.3 billion by 2028.

**Figure 1:**
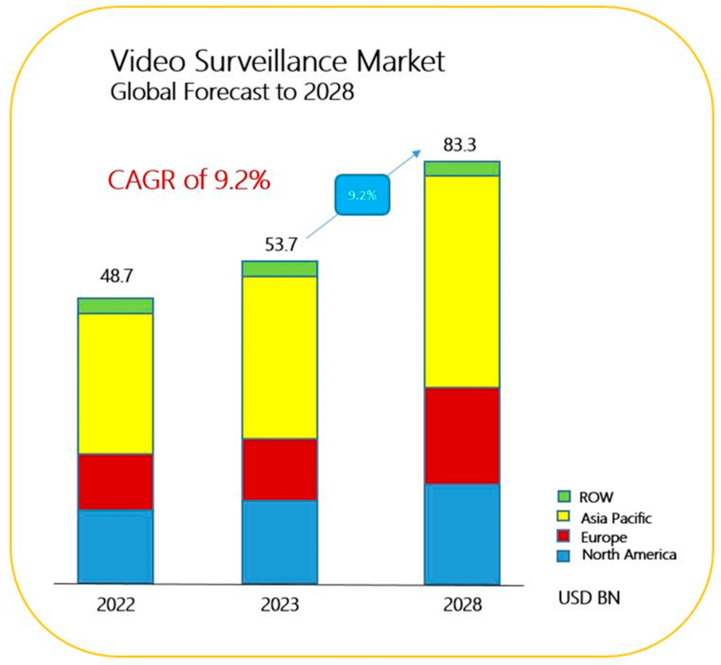
Video Surveillance Market Forecast to 2028 [1].

According to the latest report from Markets and Markets, the compound annual growth rate of the global video surveillance market from 2023 to 2028 is expected to reach 9.2%. Increasing urbanization and the trend toward smart cities have created a need for advanced IVS systems. Critical infrastructure, including transportation hubs and public utilities, require robust surveillance systems to ensure safety and operational efficiency. The IVS market is expected to reach US$83.3 billion by 2028 (Figure 1) [1]. Given the enormous business opportunities and growth potential in the video surveillance industry, this study examined key developments in the digital surveillance industry.

Web of Science database is a platform consisting of several literature search database designed to support scientific and scholarly research. Web of Science Core Collection is premier resource on the platform and includes more than 21,000 peer-reviewed, high-quality scholarly journals published. In total, 5111 articles about Video Surveillance were included for this paper. Many studies, have conducted video surveillance for a literature review on science or technology. 

This study reviewed the literature on IVS technologies to examine how the field has developed over time. Articles were retrieved from the Web of Science (WOS) academic database and analyzed using main path analysis (MPA) to examine relevant developments, theories, and trends in the field of IVS. Furthermore, cluster analysis and text mining were performed. The objectives of this study were as follows:

1. MPA was performed to identify developmental trends in the field of IVS over time.
2. Cluster analysis was performed to group IVS articles and identify main topics.
3. Text mining was performed to identify relevant key words, and growth curves were used to predict growth in topics.

### 1.1. Identifying Core Academic Literature

#### 1.1.1. Identifying Intelligent Video Surveillance

IVS systems are surveillance systems that involve the use of a large number of security cameras. These systems can integrate and analyze data from multiple cameras and perform surveillance-specific actions, such as generating warning notifications. IVS is interdisciplinary, combining electronics (sensing equipment), pattern recognition, computer vision, machine learning, and network technologies. IVS can be implemented in various settings, has a range of applications, and combines fifth-generation technologies, artificial intelligence, and the Internet of things. IVS is used in urban settings, agriculture, medicine, and transportation [2].

The seven common functions of IVS are as follows [3]:

1. Image and video analysis.
2. Construction cost reduction.
3. Alerts and notifications.
4. Cloud application.
5. Unlimited transmission distance.
6. Mobile surveillance.
7. Flexible expansion of the system.

IVS has applications in cities, agriculture, medicine, and transportation. Only a handful of literature reviews have assessed developments in IVS research. Furthermore, the analytical methods used in articles on IVS have limitations. In summary, the field of IVS has not been comprehensively analyzed.

Given the aforesaid considerations, this study reviewed the literature on IVS research by organizing articles on the basis of publication year and topic popularity. The articles were retrieved from the WOS database. Accordingly, the top 20 fields in IVS were identified to examine advancements in the field and provide recommendations for future research.

#### 1.1.2. Literature on Main Path Analyses

Many studies have conducted main path analyses or key-route main path analyses for a literature review on science or technology. Fontana et al. [4]; Verspagen [5]; and Mina and Consoli [6] employed main path analyses to identify the trajectory of technology. Calero-Medina and Noyons [7]; Strozzi and Colicchia [8]; Harris et al. [9]; Chuang et al. [10]; Bekkers and Martinelli [11] and LucioArias and Leydesdorff [12] conducted a main path analysis to investigate changes in technology. Bhupatiraju et al. [13]; Yan et al. [14]; and Su et al. [15] performed main path analysis to review literature in various disciplines. Li [16] conducted a main path analysis to simplify a massive number of patent verdicts. Li [17] also performed a main path analysis to identify key verdicts and observe trends in patent rights abuse from 1916 to 2016.

## 2. Materials and Methods

### 2.1. Data Source

The study searched the WOS database using the following terms ⸢TS = (“Intelligent Monitor” OR “Intelligent Surveillance” OR “Security cameras” OR “Surveillance cameras” OR “Smart monitoring” OR “surveillance monitor” OR “Closed-circuit television” OR “video Surveillance”)⸥. This search yielded 6498 articles. Articles were excluded if they lacked information on author, topic, or year of publication. In total, 5111 articles were included for further analysis.

### 2.2. Main Path Analysis

**Figure 2:**
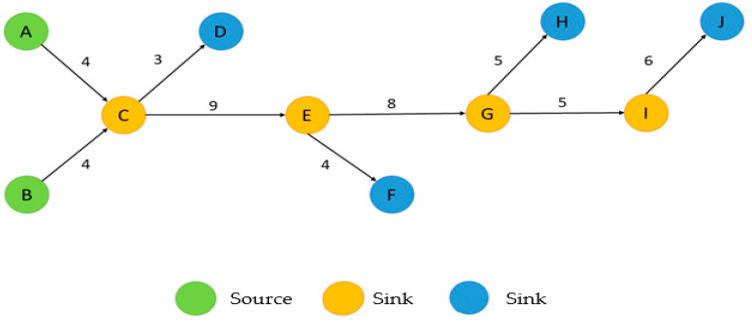
Calculation of weights by using SPLC.: The number of all possible paths from the end of the line to the sink is then calculated, and the two aforementioned numbers are multiplied to calculate the final weight of all the lines.

MPA was proposed by Hummon and Dereian [18] to analyze developments in deoxyribonucleic acid theories. MPA involves analyzing citation networks and quantifying citations of books, articles, and patents. MPA calculates the weight of each connection from the origin (source of academic articles) to the destination (sink of academic articles), then uses the weight of each path to identify the main path. This study followed the suggestions of Liu and Lu [19] and adopted two methods, namely global MPA and key-route MPA. Liu and Lu provided empirical evidence demonstrating that the search path link count (SPLC) method is superior to the search path count and search path node pair methods. They also demonstrated that MPA can effectively uncover knowledge diffusion. The SPLC weight algorithm extracts a line from the network and calculates the number of possible paths from the origin, through the nodes, to the end of the line. The number of all possible paths from the end of the line to the sink is then calculated, and the two aforementioned numbers are multiplied to calculate the final weight of all the lines (Figure 2).

### 2.3. Basic Statistics Analysis of Journals and Authors

Regarding journal statistics, each journal’s name, publication dates, and g-index and h-index values were obtained. For author statistics, each author’s name, publication dates, and g-index and h-index values were obtained.

Leo Egghe (2006) [20] proposed the g-index metric, which indicates that after academic articles and research results have been arranged in decreasing order of the number of their citations, the top g number of articles have received at least g2 citations. Hirsch (2005) [21] proposed the h-index metric, which indicates that an author has h number of articles and each article has been cited at least h times.

This study used the g-index for analysis and complemented the analysis with the h-index to assess the influence of journals in an academic field and the contribution made by authors. Therefore, this study listed the top 20 influential journals for IVS and the top 20 influential authors for IVS.

### 2.4. Growth Curve Analysis

Articles were retrieved from the WOS database. Data were analyzed and expected growth curves were drawn using Loglet Lab. The *y*-axis was the cumulative number of IVS articles, and the *x*-axis was the year. The final growth curve could predict the growth stage and maturity stage of the field of IVS.

### 2.5. Cluster Analysis

Cluster analysis was used to group similar articles, and key words were used to name each group. The Girvan–Newman algorithm was used to perform cluster analysis (Girvan & Newman, 2002) [22]. Its steps are as follows:

1. Calculate the betweenness in the network. Select two random nodes. The total number of shortest paths that pass through the two nodes is the number of edges between the two nodes.
2. Eliminate the path that has the largest betweenness.
3. Calculate the modularity of separated clusters. If no new clusters are separated, repeat steps 1 and 2 until all the paths have been eliminated. The modularity compares the strength of the associations between nodes within clusters and between nodes inside and outside clusters.
4. Select the grouping with the largest modularity. This is the optimal grouping of the cluster analysis.

### 2.6. Word Clouds

After cluster analysis, the titles and abstracts of articles in each group were analyzed, the frequency of each key word was calculated, and the results were presented in word clouds. Prepositions and articles were excluded from calculations. Key word frequencies were ranked, and groups were named using key words.

## 3. Results

### 3.1. Data Statistics

Papers related to IVS technology research were collected from the Web database. The keyword “Surveillance” was used to select papers to ensure the collected papers were relevant. The references, publication years, authors, and page numbers of 5284 papers were obtained. erroneous data, such as garbled text, blanks, and anonymous authors, were combined, 5111 papers remained. Because the Web of Science database can present the citation relationship between papers, this database is suitable the development trends of scientific territories.

**Figure 3:**
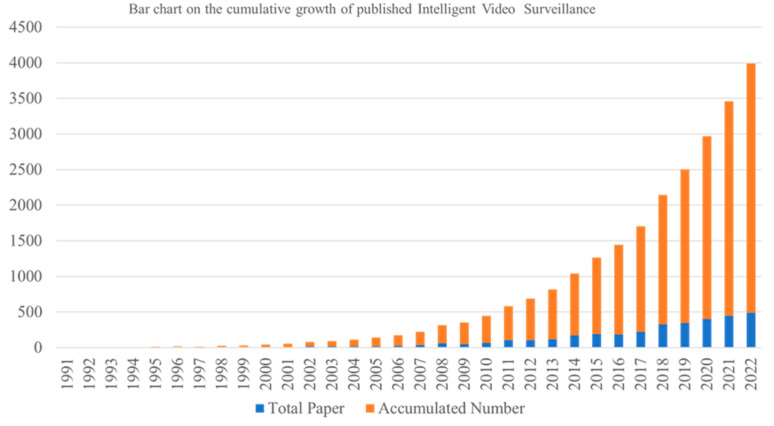
Number of articles published in field of Intelligent Video Surveillance.

The cumulative number of articles published per year is shown in Figure 3. The period spans 1991 to 2022. The blue and orange bars represent the number of articles published each year and the cumulative number of IVS articles, respectively. Regarding statistics, data on the publication period, journal name, and journal g-index were compiled. Regarding researcher data, statistics on the author, publication and g-index and h-index were compiled. Of these indices used significance of a journal or author to the IVS territories. the g-index was employed indicator and the h-index was used as the secondary indicator for separately the top 20 influential journals and authors in the IVS territories. The number of articles published each year increased slowly starting from 1995, and in 2011, the annual increment of the number of published articles increased considerably. From 2012 to 2022, the number of articles published per year exceeded 100.The number of articles published per year was highest in 2022, suggesting that the field of IVS is developing and receiving more attention.

### 3.2. Journal Statistics

The top journal was IEEE Transactions on Circuits and Systems for Video Technology, which published 116 articles (Table 1) on video collection, display, processing, filtering, conversion, synthesis, compression, transfer, communication, network, storage, retrieval, search, and hardware and software design and implementation. The high g-index value of this journal demonstrates its importance in the field of IVS. The journal with the second-highest ranking was IEEE Transactions on Image Processing, which published 60 articles explaining new theories, algorithms, and architectures related to the formation, capture, processing, communication, analysis, and display of videos and multidimensional signals. The journals that ranked third, fourth, and fifth were Pattern Recognition Letters, Pattern Recognition, and Computer Vision and Image Understanding, respectively. These journals published articles on image information and computer vision.

### 3.3. Academic Literature and the Overall Development Trajectory of IVS

**Figure 4:**
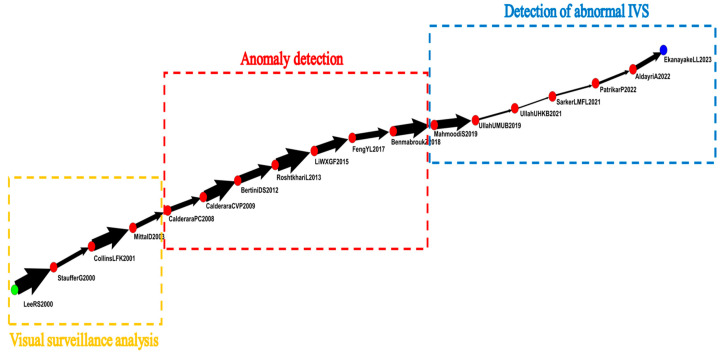
Relational diagram for global main path of academic articles.

Global MPA results are shown in Figure 4. The green and blue nodes represent the sources and sinks of articles, respectively. Each node represents an article, and directional arrows, which represent the flow of knowledge, connect the nodes. Each node has a code beside it. The code includes the name of the first author, the initials of other authors, and the year of publication. Lowercase letters were added to distinguish repeated codes.

The main path of the field of IVS is shown in Figure 4. The main path of the citation network had the highest weight and consisted of 18 nodes. Because each node represented an article, this study briefly introduced the 18 articles on the global main path.

Lee al. (2000) explored the automatic construction of a comprehensive and independent image framework. They used the videos of multiple cameras to analyze different types of videos [23]. Collins et al. (2001) integrated multicamera surveillance systems with the objective of automatically collecting and distributing real-time information to improve situational awareness among decision-makers [24]. Mittal et al. (2003) used multiview video systems to perform video segmentation tasks and to detect and track individuals [25].

Calderara (2008) and Simone et al. (2009) proposed methods for solving the problem of overlapping fields of view in multicamera systems. They proposed a complete video system that performed image segmentation and believed that video surveillance was a crucial component of intelligent transportation systems [26,27]. Marco et al. (2012) used video analysis technology in various applications, including detection of abnormal events and in surveillance systems [28]. Mehrsan et al. (2013) proposed a method that uses videos as training samples to effectively detect suspicious events in videos [29]. Nannan et al. (2015) proposed a novel anomaly detection method that uses video surveillance. They used Gaussian process regression to identify abnormal events, investigated the effects of occlusion, and used supplemental information from previous frames to perform anomaly detection [30]. Yachuang et al. (2017) believed that the detection of abnormal events by IVS is essential, particularly for crowded settings [31]. According to Ben et al. (2018), the two main components of video surveillance systems are behavior representation and modeling. They used feature extraction and relevant technologies to describe behavior representation and provided classification methods and frameworks for behavior modeling [32].

According to Ullah et al. (2019), surveillance cameras enable the collection of large amounts of data [33]. According to Mahmoodi et al. (2019), IVS can be used for violence detection. As the demand for video surveillance systems that can automatically detect violence increases, current violence detection methods should be researched and improved [34]. Mohammad et al. (2021) and Waseem et al. (2021) believed that automatic anomaly detection is crucial when video surveillance monitors the environment [35,36]. Patrikar et al. (2022) investigated IVS-based anomaly detection methods [37]. According to Amnah et al. (2022), the increasing prevalence of CCTV has accentuated the importance of detecting anomalies in videos of crowds through IVS. Such detection tasks are challenging because personnel are required to dedicate considerable time and continuous attention to effectively identify abnormalities in the large amount of videos captured by CCTV systems [38]. According to Ekanayake et al. (2023), crowd density and anomaly detection are popular topics of video surveillance, particularly people-oriented actions and activity-based motions. These topics focus on attention-oriented classification systems based on deep learning to recognize basic activities in public venues [39].

## 4. Discussion

### 4.1. Development Trajectory of Intelligent Video Surveillance

This study performed MPA to identify the main path with the highest weight. Next, key-route MPA was performed to identify the following three periods. Global MPA was performed to find the main path with the highest weight, and key-route MPA was performed to observe interactions and associations between paths. The three types of MPA revealed the development of the field of IVS.

1. Visual surveillance analysis (2000–2003): development strategy of IVS in visual surveillance. Articles during this period focused on key elements related to the development of IVS systems, such as motion tracking, camera coordination, activity classification, and event detection.
2. Anomaly detection (2008–2018): analysis of intelligent anomaly detection. Articles during this period focused on anomaly detection methods and proposed video analysis techniques to automatically analyze videos and immediately alert users of abnormal activities. Anomaly detection can supervise other surveillance tasks. The articles also proposed new methods for anomaly detection by video surveillance.
3. Detection of abnormal (2019–2023): effects of abnormalities detected from intelligent surveillance. Articles during this period explored the effects of abnormal intelligent surveillance. They believed that the detection of abnormal intelligent surveillance and the protection of personal privacy are both essential and that system operations and personal privacy can be maintained.

**Figure 5:**
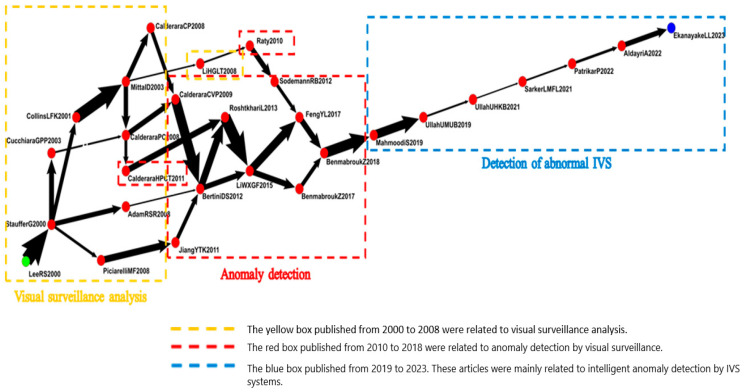
Key-route main path of IVS articles.

This study drew the key-route main path to ensure that influential articles were not omitted. Figure 5 demonstrates the relationship between multiple paths and reveals the development of IVS articles at different times.

In Figure 5, the yellow box reveals that the IVS articles published from 2000 to 2008 were related to visual surveillance analysis. In general, these articles discussed the automatic detection and tracking of multiple people in high-density settings.

The red box demonstrates that the IVS articles published from 2010 to 2018 were related to anomaly detection by visual surveillance. These articles explained that as the number of indoor and outdoor cameras increased, the demand for the detection of abnormalities among moving objects in videos increased as well.

The blue box displays the IVS articles published from 2019 to 2023. These articles were mainly related to intelligent anomaly detection by IVS systems. To automatically detect abnormalities, improve problems related to artificial methods, and increase effectiveness, these articles proposed identification frameworks that incorporated convolutional neural networks (CNNs) to accurately detect abnormalities in videos. In general, this path focused more on deep learning.

The present study observed the development of IVS articles and discovered that most of them were related to video surveillance analysis, anomaly detection by video surveillance, and intelligent anomaly detection. To understand other fields, this study grouped similar articles in other fields, analyzed the titles of articles in each group, and created word clouds. The groups were named by using the study that was cited the most in each group. This study reviewed the titles of the articles to form word clouds. After cluster analysis, six groups were created, some of which were related to applications of IVS systems (Table 2). To further explore each group, the articles in each group were analyzed, SPLC weights were calculated, and main paths were drawn to ensure that each group included influential IVS articles.

### 4.2. Cluster Analysis of IVS

An edge-betweenness cluster analysis yielded 20 clusters, and studies in the top 6 clusters, namely the effects of IVS. Table 2. presents the themes, number of studies, keywords, and word clouds for these 6 clusters. Keywords were ranked by their frequency (numbers in parentheses) in titles; for example, “detection” appeared an average 0.01 times in the first cluster. The studies in each cluster were analyzed to determine the main path of each cluster and the research direction of each main path. The literature growth trend charts revealed that the number of studies in the literature increased in all six clusters.

The aforementioned MPA divided the field of IVS into several groups to gain insights into the key topics. A total of 6 groups were identified. The top six groups were named using the key words collected by Wordle, and they were IVS in video camera, IVS in background modeling, IVS in closed-circuit television CCTV, IVS in multiple cameras, IVS in person reidentification, and IVS in privacy, security, and protection. The articles within each group were analyzed to obtain their main paths and to investigate the development of IVS research in each group.

#### 4.2.1. IVS in Video Cameras

**Figure 6:**
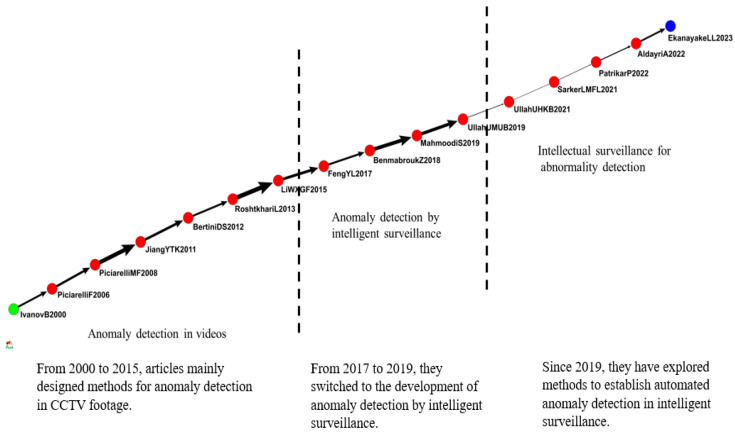
IVS in video cameras.

The first group included 573 articles related to video cameras (Figure 6). The main path of this group extended from 2000 to 2023 and included 16 articles that discussed the effects of video cameras on IVS.

Ivanov et al. (2000) discussed identification problems from two perspectives and mentioned temporal extension and interaction activities for the detection and identification of multiple videos [40]. Piciarell et al. (2008) proposed the detection of abnormal events that differed from the norm. They used trajectory analysis for anomaly detection, particularly for video and traffic surveillance [41]. Jiang et al. (2011) suggested vertical screen perception for anomaly detection. They tracked all moving objects in videos and considered the spatiotemporal context at three levels, namely the point anomaly of video objects, the sequential anomaly of object trajectories, and the co-occurrence anomaly of multiple video objects [28].

Bertini et al. (2012) explored an anomaly detection and positioning method applied in video surveillance to collect statistics in a dynamic setting and external spatiotemporal features [42]. Li et al. (2015) proposed an anomaly detection method for video surveillance of crowded settings. Their method was called an automated statistical learning framework and was based on the analysis of the layout of volumes of three-dimensional objects in spatiotemporal videos. The method could effectively detect abnormalities and precisely locate abnormal regions [43].

According to Feng et al. (2017), detection of abnormal events in video surveillance is crucial, particularly for complex settings. They used a deep learning network for image classification (PCANet) and extracted appearance and motion features from three-dimensional gradients to model the events. They constructed a Gaussian mixture model (GMM) from normal events that they observed. A deep GMM is an expandable deep generative model, and Feng et al. stacked multiple GMMs together so that their method could use relatively few parameters to achieve competitive performance [31]. Ben Mabrouk et al. (2018) investigated the two main components of video surveillance systems, namely behavior representation and behavior modeling. They reviewed the feature extraction of behavior representation and described relevant techniques, and provided classification methods and frameworks for behavior modeling [32]. Waseem et al. (2021) proposed a high-efficiency intelligent anomaly detection framework based on deep features. The framework extracted features from frames and was valuable for capturing abnormalities [36].

Mohammad et al. (2021) introduced and analyzed methods for video anomaly detection and the reliability of such methods. They proved the high sensitivity of anomaly detection in a variety of circumstances [35]. Patrikar et al. (2022) developed various methods for anomaly detection in IVS. Anomaly detection is considered a key temporal application of computer vision. Edge devices and specialized methods are used for automated anomaly detection [37]. The use of CCTV has become more common in smart cities. According to Amnah et al. (2022) and Ekanayake et al. (2023), for crowd anomaly detection, IVS is essential. Articles related to the detection of human behavior include methods that detect abnormal crowd behaviors [38,39].

Figure 6 demonstrates that from 2000 to 2015, articles mainly designed methods for anomaly detection in CCTV footage. From 2017 to 2019, they switched to the development of anomaly detection by intelligent surveillance. Since 2019, they have explored methods to establish automated anomaly detection in intelligent surveillance.

#### 4.2.2. IVS in Background Modeling

**Figure 7:**
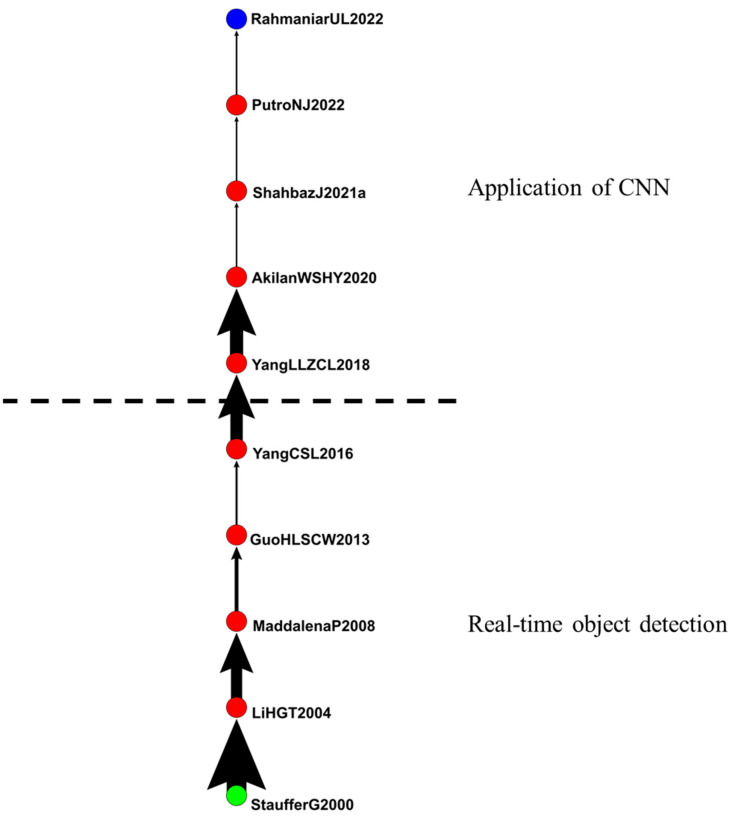
IVS in background modeling.

The second group included 436 articles related to background modeling (Figure 7). The main path extended from 2000 to 2022 and included 10 articles that discussed the effects of background modeling on IVS.

Stauffer et al. (2000) and Maddalena et al. (2008) explored the detection and tracking of moving objects by artificial intelligence. The key elements of their method were motion tracking, camera coordination, activity classification, and event detection. They focused on motion tracking and demonstrated how to use the motions observed to learn activities at various learning points [44,45]. Li et al. (2004) proposed a novel algorithm for detecting foreground objects in a complex environment. The algorithm consisted of change detection, change classification, foreground segmentation, and backend maintenance and was used to arrange the order of interesting images in various environments such as offices and public buildings [46].

According to Guo et al. (2013), the detection of moving objects is a fundamental step of IVS. They proposed a solution that provided highly precise and effective processing that satisfied the need for real-time detection of moving objects [47]. According to Yang et al. (2013 and 2018), background information processing, such as object detection and scene understanding, is crucial for video surveillance. They proposed a pixel-to-model method for background modeling and for restoring monitored settings [48,49]. According to Akilan et al. (2020), foreground and background segmentation in videos is useful in intelligent transportation and video surveillance. Current algorithms are mostly based on conventional computer vision techniques, but the newest solution uses deep learning models that focus on image classification [50].

In their study, Shahbaz et al. (2021) highlighted the security risks associated with unauthorized access to restricted areas. To address this issue, they suggested integrating IVS with a sterile zone monitoring algorithm. However, implementing such an algorithm comes with its own set of challenges, including double cameras (color and infrared), dynamic background, lighting variations, camouflage, and static foreground objects. To address these challenges, Shahbaz et al. proposed an improved change detector algorithm [51]. Putro et al. (2022) proposed a high-efficiency face detection algorithm that uses lighting to precisely locate faces [52]. According to Rahmaniar et al. (2022), head posture estimation is used in several IVS systems, such as human behavior analysis, intelligent driver assistance, and visual warning and monitoring systems. These systems require precise alignment and prediction of head movements. Rahmaniar et al. proposed a method to estimate head postures using facial conditions, such as occlusion or challenging viewpoints [53].

Figure 7 reveals that from 2000 to 2016, articles focused on real-time object detection. Since 2018, articles have gradually changed their attention to the application of CNN in IVS.

#### 4.2.3. IVS in Person Reidentification (PReID)

**Figure 8:**
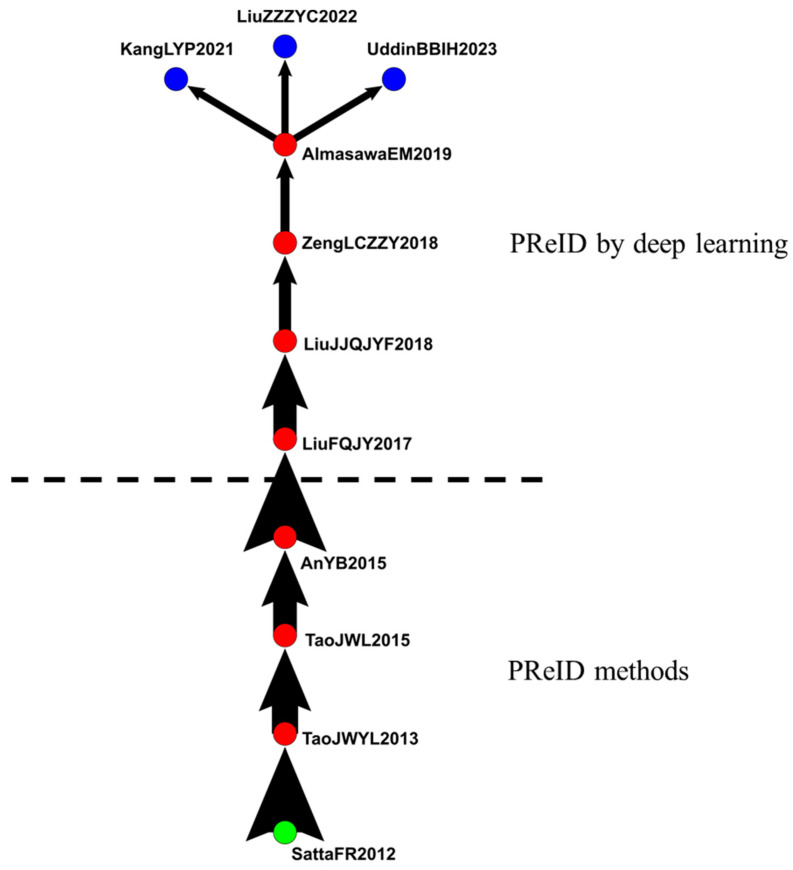
IVS in person reidentification.

The third group included 207 articles related to PReID (Figure 8). The main path extended from 2012 to 2023 and included 11 articles that discussed the effects of PReID in IVS.

Satta et al. (2012), Tao et al. (2013 and 2015), and An et al. (2015) discussed PReID, focusing on matching people at different times and locations. The computer vision of PReID includes the identification of individuals who have previously passed through the surveillance camera network [54,55,56,57]. Liu et al. (2017) proposed a novel model based on soft attention called the end-to-end comparative attention network, which was specifically designed for PReID tasks [58]. Liu et al. (2018) stated that the PReID in videos is a core function of security and video surveillance. They proposed a new accumulative motion context network for this crucial issue [59].

Zeng et al. (2018) mentioned that PReID is a new task of IVS and is closely associated with many actual applications [60]. Almasawa et al. (2019), Kang et al. (2021), Liu et al. (2022), and Uddin et al. (2023) argued that PReID plays a crucial role in IVS and has diverse applications in public safety. They proposed using deep learning to improve PReID systems, and their articles are essential for different applications of computer vision [61,62,63,64].

Figure 8 reveals that from 2012 to 2015, articles mainly discussed the development of PReID in IVS. Since 2017, articles have gradually switched to the application of deep learning in PReID.

#### 4.2.4. IVS in Closed-Circuit Television

**Figure 9:**
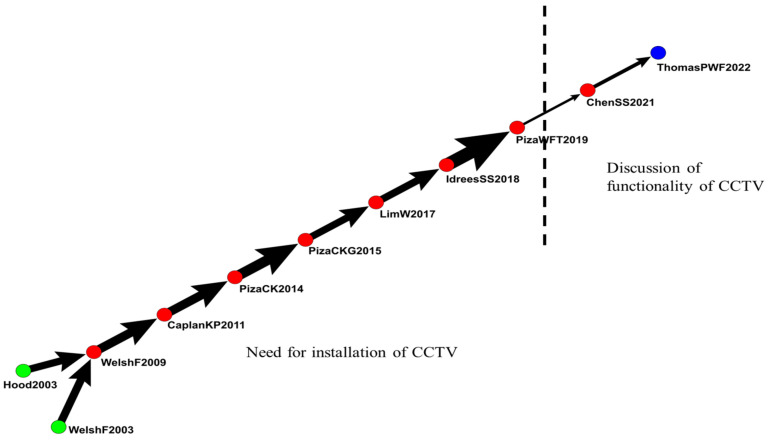
IVS in closed-circuit television.

The fourth group included 177 articles related to CCTV (Figure 9). The main path extended from 2003 to 2022 and included 11 articles that discussed the effects of CCTV in IVS.

Welsh, BC et al. (2003) systematically reviewed articles investigating the effects of CCTV on crime at public venues. They performed a targeted and comprehensive search on published and unpublished articles [65]. Welsh et al. (2009) and Caplan et al. (2011) performed the latest systematic review and meta-analysis on the effects of CCTV on crime at public venues [66,67]. Piza et al. (2014 and 2015) explored whether environmental features changed in accordance with the type of crime. They discovered that the effect of the environment on crime rates differed by the type of crime. For example, CCTV is associated with less crime, less violent crime, and less motor vehicle theft, and stationary objects are associated with the increase of CCTV occlusion and motor vehicle theft and the decrease of violent crime and robbery [68,69].

Lim et al. (2017) and Piza et al. (2019) discovered limited evidence supporting the effectiveness of CCTV in reducing crime. Furthermore, they observed that the effectiveness was influenced by the underlying crime rate [70,71]. Idrees et al. (2018) introduced and discussed computer vision from the perspective of law enforcement. Their research is valuable for law enforcement personnel who monitor large camera networks and who are responsible for upgrading computer vision systems [72]. Chen et al. (2021) observed that a drastic increase in the number of surveillance cameras did not provide a crime deterrent effect nor evidence for investigations [73]. Thomas et al. (2022) mentioned the global expansion of CCTV programs and used systematic review methods and meta-analytic techniques to investigate the effects of CCTV programs on crime rates in different countries [74].

Figure 9 reveals that from 2003 to 2019, articles mainly discussed the need to install CCTV in IVS. Since 2019, articles have begun to change their focus on the functionality of IVS.

#### 4.2.5. IVS in Privacy, Security, and Protection

**Figure 10:**
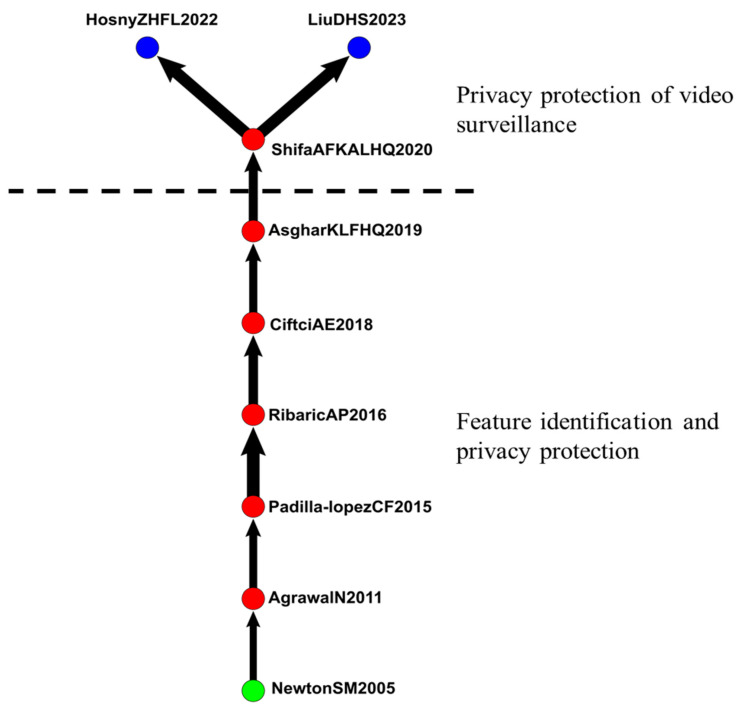
IVS in privacy, security, and protection.

The fifth group included 170 articles related to privacy, security, and protection (Figure 10). The main path extended from 2005 to 2023 and included 9 articles related to the effects of privacy, security, and protection in IVS.

Newton et al. (2005) introduced an algorithm that protected the privacy of video surveillance data. The algorithm de-identifies faces to retain facial features without reliable identification of individuals [75]. According to Agrawal et al. (2011), the improvement of cameras and network technologies has facilitated the capture of large amounts of video data and extensive video sharing. However, automated methods are required to de-identify individuals in videos [76].

Ramon et al. (2015) explored methods to protect individual privacy in image data. Their main contribution was proposing visual privacy protection methods [77]. Ribaric et al. (2016), Ciftci et al. (2018), and Asghar et al. (2019) discussed the concept of privacy and the relationship between privacy and data protection. They also investigated privacy protection designs and techniques for multimedia data and used a technological perspective to understand visual privacy protection [78,79,80].

Shifa et al. (2020) stated that video surveillance is often used for real-time anomaly detection and automated video analysis. The videos captured by real-time surveillance cameras often include identifiable personal information, which could include the location of surveillance and other sensitive data, and must be protected [81]. Hosny et al. (2022) proposed a new method to protect the privacy of individuals in surveillance videos. Their simulation results and safety analysis confirmed the effectiveness of their method for protecting the privacy of individuals in surveillance videos [82]. Liu et al. (2023) argued that rapid technological development increased the number of video surveillance equipment in family settings, and the importance of video privacy protection facilitated the development of different video privacy protection methods [83].

Figure 10 reveals that from 2005 to 2009, articles mainly explored feature identification and privacy protection in IVS. In 2020, articles started to discuss privacy protection in video surveillance.

#### 4.2.6. IVS in Multiple Cameras

The sixth group included 147 articles related to multiple cameras (Figure 9). The main path extended from 2000 to 2022 and included 10 articles related to the effects of multiple cameras in IVS.

Lee et al. (2000) discussed the automatic construction of a comprehensive image-independent framework that used the videos of multiple cameras to model activities on a large scale [23]. Collins et al. (2001) and Snidaro et al. (2003) aimed to automatically collect videos and distribute real-time information by using visual-based surveillance systems and autonomous vehicles to improve situational awareness among security providers and decision-makers [24,84].

Wang, Xiaogang (2013) and Kenk et al. (2015) reviewed the latest developments of relevant technologies from the perspective of computer vision and model identification. They proposed an integrated solution for the reidentification problem of distributed intelligent cameras [85,86]. Iguernai et al. (2019) introduced the multicamera tracking of objects and summarized and classified some common methods [87].

Olagoke et al. (2020) evaluated articles that investigated the physical layout, calibration, algorithm, advantages, and disadvantages of multicamera systems [88]. Liu et al. (2021) explored the storage, application, and development of wireless video surveillance systems [89]. Yu et al. (2021 and 2022) suggested using cameras connected to the Internet to monitor individuals, families, and environments. They also proposed solutions for improving privacy and storage [90,91].

**Figure 11:**
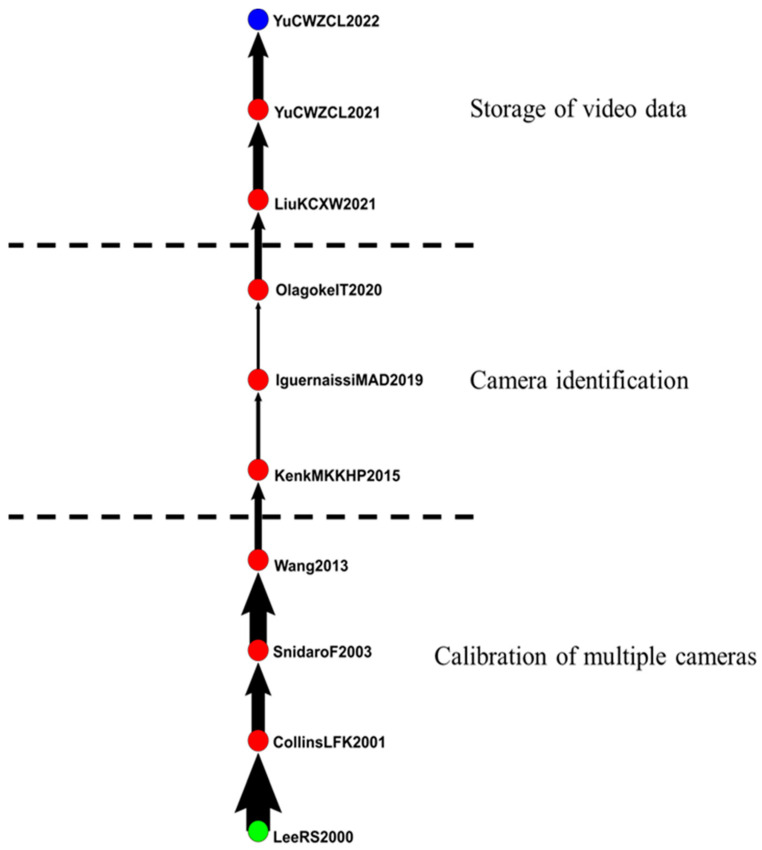
IVS in multiple cameras.

Figure 11 reveals that articles discussed the calibration of multiple cameras from 2000 to 2013, discussed camera identification from 2015 to 2020, and began exploring the storage of video data in 2021.

#### 4.2.7. Emerging Areas and Potential Opportunities in Other Applications

This study collected more than 77 IVS articles and discovered three additional groups (Table 3). The three groups are arranged by the number of articles published and represent the application of IVS in action recognition, face recognition, and cloud computing. Because these groups contain few articles, not all journals appeared on the main paths of these groups. In addition, journals that performed literature reviews for these articles focused solely on the development of specific fields and made significant contributions to those fields. However, this study only considered influential journals and fields with large numbers of published articles (Table 3). Journals that performed literature review focused on the development of specific types of technology, such as improving recognition and analysis of different actions in videos and enhancing big data analysis. They also developed automatic detection, classification, and analysis of objects in videos to create more precise surveillance and analysis. Therefore, if these journals continue their research, they could improve the performance of IVS in the future.

### 4.3. Analysis of Growth Curve of IVS

**Figure 12:**
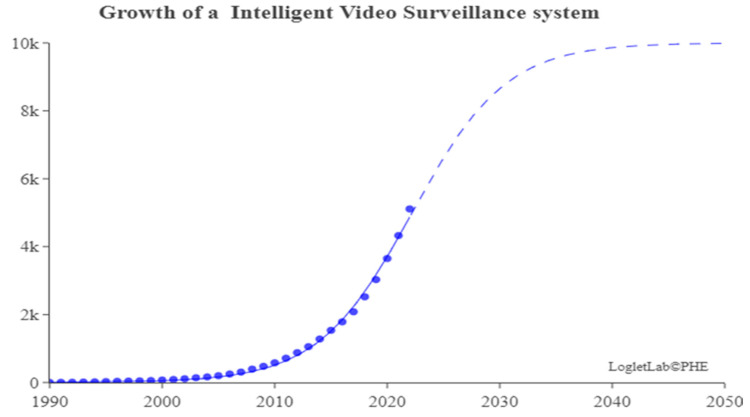
Growth curve of number of articles.

Loglet [92] analysis involves the decomposition of growth and diffusion patterns into S-shaped logistic components. The decomposition is roughly analogous to wavelet analysis, popular for signal processing and compression. In the easiest cases, a loglet appears as a single S-shaped curve. This study adopted a Logistic growth model. Loglet Lab was used to depict the growth curve of IVS and predict its maturity stage, growth stage, peak, and turning point. The dotted line in Figure 12 represents the expected total cumulative number of published articles. The solid line and the circles represent the actual total cumulative number of published articles. The results demonstrated that 2020 was the turning point of the growth curve. The curve is expected to reach the mature stage by 2035, at which time the maximum cumulative number of published articles is expected to exceed 6000. The results also indicate that the field of IVS is still in its growth stage and is 15 years away from its maturity stage.

## 5. Conclusions

This study used cluster analysis and text mining to identify the top six groups of 5111 articles and analyze fields related to IVS. The six main groups were IVS in video cameras, IVS in background modeling, IVS in PReID, IVS in CCTV, IVS in privacy, security, and protection, and IVS in multiple cameras. The conclusion regarding the future development focus of the six topic groups are described as follows:

1. IVS in video cameras: The detection of abnormal events in intelligent surveillance is crucial. The accurate determination of abnormal events in complex settings is particularly important.
2. IVS in background modeling: The application of CNN in IVS.
3. IVS in PReID: The usage of deep learning to improve accuracy and efficiency in PReID.
4. IVS in CCTV: The intellectualization of CCTV.
5. IVS in privacy, security, and protection: The protection of personal privacy in surveillance systems.
6. IVS in multiple cameras: The storage of data from multiple cameras.

The global MPA, key-route MPA, and cluster analysis of the six groups demonstrated that although the groups were different, they were still associated. The “visual surveillance analysis”, “anomaly detection”, and “detection of abnormal intelligent surveillance” of the global main path and key-route main path corresponded with the “IVS in video cameras”, “IVS in background modeling”, and “IVS in PReID” groups, indicating that these fields had a certain degree of influence on IVS. Furthermore, the groups demonstrated special applications, particularly among IVS in privacy, security, and protection. The importance of video privacy and protection facilitated the development of video privacy and protection methods. On the basis of the aforementioned analysis, this study suggests that further research into privacy, security, and protection is warranted.

This study comprehensively explored developments in the field of IVS and elaborated on key implications.

## Figures and Tables

**Table 1:** Top 20 journals in the field of IVS.

G-Index Ranking	Journal	*g*-Index	*h*-Index	Active Years	Papers after 2000
1	IEEE Transactions on Circuits and Systems for Video Technology	66	40	1998~2022	116
2	IEEE Transactions on Image Processing	60	29	2003~2022	60
3	Pattern Recognition Letters	50	23	2003~2022	65
4	Pattern Recognition	48	22	2006~2023	62
5	Computer Vision and Image Understanding	43	23	2006~2023	43
6	IEEE Transactions on Pattern Analysis and Machine Intelligence	42	28	2000~2022	42
7	Neurocomputing	41	22	2008~2022	65
8	IEEE Transactions on Intelligent Transportation Systems	39	23	2011~2022	50
9	Multimedia Tools and Applications	39	22	2010~2023	211
10	Image and Vision Computing	39	18	2002~2022	39
11	IEEE Access	37	25	2013~2023	159
12	Sensors	37	24	2012~2023	162
13	Expert Systems with Applications	35	21	2008~2023	49
14	IEEE Transactions on Multimedia	34	21	2005~2022	34
15	IEEE Transactions on Information Forensics and Security	31	18	2013~2023	31
16	Machine Vision and Applications	28	18	2006~2022	47
17	Automation in Construction	26	16	2001~2022	26
18	IEEE Internet of Things Journal	25	12	2018~2023	26
19	IEEE Sensors Journal	23	13	2002~2022	29
20	Journal of Visual Communication and Image Representation	21	14	2007~2022	47
				Total	1363

**Table 2:** Topics, number of articles, growth curves, and word clouds.

Theme	Keywords	Growth Curve	Word Cloud
Group1 (573 papers) ISSs in Video Camara	Detection (0.01) Surveillance (0.01) Video (0.01) Deep (0.002) Intelligent (0.002) System (0.003) Behavior (0.004) Data (0.004) Performance (0.004) Method (0.005)	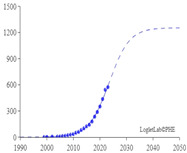	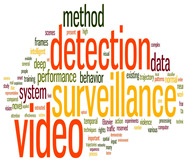
Group2 (436 papers) ISSs in Background Modeling	Model (0.01) Objects (0.01) Algorithm (0.01) Background (0.012) Dynamic (0.002) Learning (0.002) Real-time (0.002) Segmentation (0.002) Analysis (0.003) Applications (0.003)	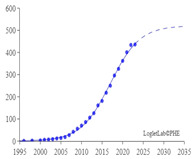	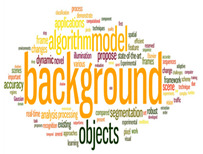
Group3 (207 papers) ISSs in Person Re-Identification (PReID)	Person (0.01) Re-identification (0.02) Feature (0.01) Different (0.01) Datasets (0.01) Results (0.004) Propose (0.01) Approach (0.004) Challenging (0.003) Matching (0.003)	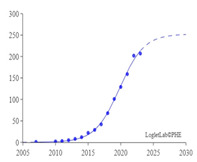	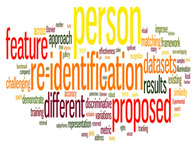
Group4 (177 papers) ISSs in Closed-Circuit Television (CCTV)	CCTV (0.015) Crime (0.01) Effects (0.01) Policy (0.01) Evidence (0.001) Safety (0.001) Control (0.002) Findings (0.003) Research (0.004) Public (0.004)	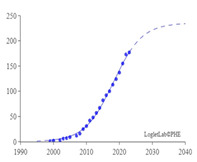	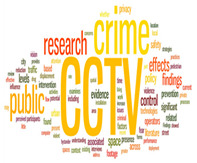
Group5 (170 papers) ISSs in Privacy security and Protection	Privacy (0.01) Protection (0.01) Security (0.01) Recognition (0.001) Devices (0.002) Framework (0.002) Smart (0.002) Storage (0.002) Encryption (0.003) Face (0.004)	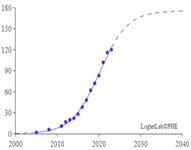	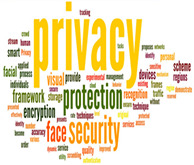
Group6 (147 papers) ISSs in Multiple Camera	Cameras (0.011) Monitoring (0.002) Network (0.002) Processing (0.002) Multiple (0.003) Problem (0.003) View (0.003) Image (0.004) Paper (0.004) Performance (0.004)	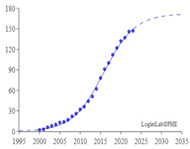	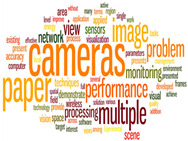

**Table 3:** Research themes ranked seventh to ninth.

	Title	Papers
7	IVS in Action Recognition 2006–2023	108
8	IVS in Face Recognition 2003–2023	98
9	IVS in Cloud Computing 2010–2023	77

## Data Availability

Data are contained within the article.
